# Distribution of entangled photon pairs over few-mode fibers

**DOI:** 10.1038/s41598-017-14955-z

**Published:** 2017-11-02

**Authors:** Liang Cui, Jie Su, Xiaoying Li, Z. Y. Ou

**Affiliations:** 1 College of Precision Instrument and Opto-electronics Engineering, Tianjin University, Key Laboratory of Optoelectronics Information Technology of Ministry of Education, Tianjin, 300072 China; 2 Department of Physics, Indiana University-Purdue University Indianapolis, Indianapolis, Indiana, 46202 USA

## Abstract

Few-mode fibers (FMFs) have been recently employed in classical optical communication to increase the data transmission capacity. Here we explore the capability of employing FMF for long distance quantum communication. We experimentally distribute photon pairs in the forms of time-bin and polarization entanglement over a 1-km-long FMF. We find the time-bin entangled photon pairs maintain their high degree of entanglement, no matter what type of spatial modes they are distributed in. For the polarization entangled photon pairs, however, the degree of entanglement is maintained when photon pairs are distributed in *LP*_01_ mode but significantly declines when photon pairs are distributed in *LP*_11_ mode due to a mode coupling effect in *LP*_11_ mode group. We propose and test a remedy to recover the high degree of entanglement. Our study shows, when FMFs are employed as quantum channels, selection of spatial channels and degrees of freedom of entanglement should be carefully considered.

## Introduction

Quantum communication has experienced rapid progress in recent years, and is developing towards a practical and mature technology^1^. Transferring high volume of quantum information between distanced places is a paramount task of quantum communication. An effective solution for the task is to encode quantum information on single photons or entangled photons, and distribute the photons over quantum channels of low losses such as optical fiber links. Previously, several examples of entangled photon pair distribution over single-mode fiber (SMF) with distances up to 200 kilometers were successfully demonstrated^2–6^. More recently, quantum key distribution and quantum teleportation have been achieved over distances of several to tens of kilometers by using metropolitan SMF networks^7–9^, which further confirms the capability of SMF network as quantum channel.

**Figure 1:**
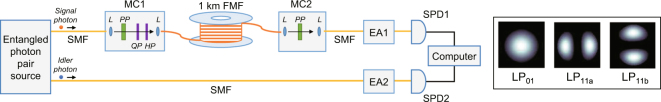
Setup for distribution of entangled photon pairs over FMF. FMF, few-mode fiber; MC, mode convertor; SMF, single-mode fiber; L, lens; PP, phase plate; QP, quarter-wave plate; HP, half-wave plate; EA, entanglement analyzer; SPD, single photon detector. Inset: the first few spatial modes of FMF.

In the past decades, multiplexing technologies in different degrees of freedom, such as time, wavelength, polarization, and phase, have been exploited to increase the information carrying capacity of SMF^10–13^. However, due to the fast growing internet traffic, the current fiber communication network based on SMFs is reaching its capacity limit^14^. Recent studies have shown that the space-division multiplexing (SDM) based on few-mode fibers (FMFs) is a promising solution to break through such a limit^15,16^. The core diameter of an FMF is slightly larger than that of an SMF, so FMFs can support a few higher-order spatial modes such as *LP*_11_ modes in addition to the fundamental mode *LP*_01_ (see inset of Fig. 1 for the mode structures). Thus the capacity of fiber network can be increased by using the multiplicity of space channels. Since practical quantum communication needs to employ fiber networks as the quantum channels, it is of great importance to evaluate whether the FMF network is capable of carrying quantum information. In a recent work, single photons and classical light were simultaneously transmitted over a 2-m-long FMF in different spatial and polarization modes^17^, which showed orthogonal spatial modes can be used for multiplexing classical and quantum channels in a short FMF. However, distribution of entangled photon pairs over long distance FMF has not been investigated yet.

In this paper, we experimentally investigate the distribution of two kinds of entangled photon pairs, that is, time-bin entangled and polarization-entangled photon pairs, over a 1-km-long two-mode FMF. By measuring and comparing the visibility of coincidence counts of entangled photon pairs before and after the distribution, we study the factors influencing the entanglement distribution for the two kinds of entanglement. The results can be employed to evaluate the capability of FMF as a long distance quantum channel for high capacity quantum communication.

## Results

Our experimental setup for studying the distribution of entangled photons over FMF is shown in Fig. 1. The entangled photon pair source is based on pulse pumped spontaneous four wave mixing (SFWM) and emits signal and idler photon pairs in the 1550 nm band, which are entangled in certain degrees of freedom such as time or polarization. The outputs of the source are coupled with SMF, so the photons are in the fundamental transverse mode, or the *LP*_01_ mode. The 1-km-long FMF (Two-mode Step-index, OFS) for entanglement distribution has a step-index core with a core diameter of ~19 *μ*m and supports transmission of *LP*_01_ and *LP*_11_ modes in 1550 nm band (see inset of Fig. 1). For both modes, the transmission loss < 0.22 dB/km.

The entanglement properties of the photon pairs are tested with two entanglement analyzers, EA1 and EA2, and two single photon detectors, SPD1 and SPD2. Both the EAs and SPDs are SMF-coupled. The EAs are constructed according to the degree of freedom of the entanglement. The electrical signals of the SPDs are acquired and processed by a computer-based data acquisition system. For the signal and idler photon pairs produced by the same pump pulse, an interference fringe in the coincidence count rate can be obtained by properly adjusting EA1 and/or EA2. The degree of entanglement is reflected in the visibility of the fringe.

As a proof of principle experiment, we only distribute the signal photons over the FMF, while the idler photons are transmitted over a short piece of SMF. This configuration simplifies the setup and data analyzing. When the signal photons are transmitted over FMF in mode other than *LP*_01_, mode conversion is needed for SMF coupling. As shown in Fig. 1, two mode converters (MCs) based on free-space phase plates (PPs) are placed at the input and output ports of the FMF, respectively. In MC1, photons from SMF are coupled into free space by an aspheric lens and then collected into FMF by another aspheric lens. The rotatable phase plate having a phase jump of *π* between two half planes is employed to convert the spatial mode of signal photons from *LP*_01_ to *LP*_11_ (see Fig. 1), while the quarter-wave plate (QP) and half-wave plate (HP) are employed to control the polarization of the passing photons. Therefore, by inserting or removing the phase plate, the signal photons can be coupled into FMF in *LP*_11_ or *LP*_01_ mode, respectively. MC2 is the reverse of MC1 but with the absence of the quarter-wave plate and half-wave plate.

### Distribution of time-bin entangled photon pairs over FMF

**Figure 2:**
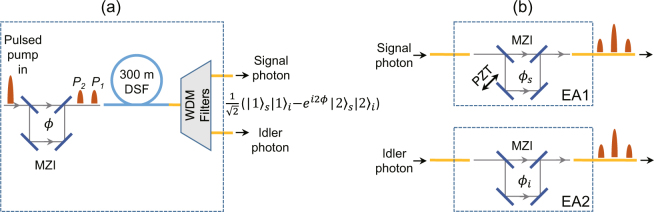
**(a)** Time-bin entangled photon pair source and **(b)** entanglement analyzers (EAs). DSF, dispersion-shifted fiber; MZI, Mach-Zehnder interferometer; WDM filters, wavelength-division multiplexing filters; PZT, piezoelectric-transducer.

Since temporal degree of freedom is not coupled with the spatial degree, we expect the case of time-bin entanglement is much simple and first distribute it over the 1-km-long FMF. The time-bin entangled photon pair source shown in Fig. 2(a) is based on pulse pumped SFWM process in a 300-m-long dispersion-shifted fiber (DSF), which is similar to the source reported in ref.^4^ In SFWM process, two photons of the strong pump are annihilated via ${\chi }_{xxxx}^{\mathrm{(3)}}$ nonlinearity to simultaneously create quantum mechanically correlated signal and idler photon pairs. In our source, the central wavelength and bandwidth of the pump pulses are 1555.61 nm and 1.2 nm, respectively. By using a free space unbalanced Mach-Zehnder interferometer (MZI), the pump pulses are decomposed into two temporally separated components, *P*_1_ and *P*_2_. The components *P*_1_ and *P*_2_ define two time slots and the time delay between the two slots is ~ 1.5 ns. *P*_1_ generates correlated photon pairs $\mathrm{|1}{\rangle }_{s}|1{\rangle }_{i}$ in the first time slot, while *P*_2_ generates photon pairs $\mathrm{|2}{\rangle }_{s}\mathrm{|2}{\rangle }_{i}$ in the second time slot. Here the subscript _*s*(*i*)_ denotes the signal (idler) photon. Since *P*_1_ and *P*_2_ are coherent, a time-bin entangled state $|{\rm{\Psi }}\rangle =\frac{1}{\sqrt{2}}{\mathrm{(|1}\rangle }_{s}\mathrm{|1}{\rangle }_{i}-{e}^{i2\phi }\mathrm{|2}{\rangle }_{s}\mathrm{|2}{\rangle }_{i})$ is produced, where $\phi$ refers to the relative phase difference between *P*_1_ and *P*_2_. The output entangled signal and idler photons are filtered by using 200-GHz-spacing wavelength-division multiplexing (WDM) filters with central wavelengths at 1560.61 nm and 1550.92 nm, respectively.

The time-bin EAs in signal and idler channels are shown in Fig. 2(b). Each EA consists of an unbalanced MZI that has the same path length difference as the MZI used to split pump pulses (see Fig. 2(a)). The relative phase difference of the MZI of EA1 (EA2) is labeled as ${\phi }_{s}$ (${\phi }_{i}$). At the output port of EA1(EA2), there will be three peaks of photon detection for photon pairs produced by each pump pulse. For the central peak, one can not distinguish whether the pair of photons are generated by *P*_1_ or by *P*_2_, thus quantum interference occurs. A piezoelectric-transducer (PZT)-driven mirror is mounted in EA1 to introduce a variation in ${\phi }_{s}$. By measuring the coincidence of the photon pairs as a function of the voltage of PZT ${V}_{PZT}$, one can obtain an interference fringe of coincidence counts as well as the degree of entanglement.

**Figure 3:**
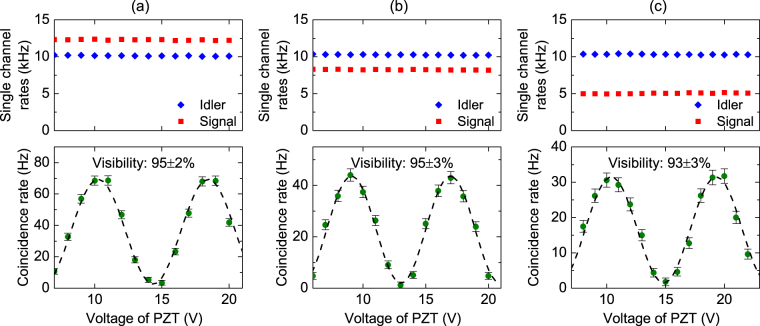
Measured single channel count rates and coincidence count rates of time-bin entangled photon pairs. The data are plotted as functions of the voltage of piezoelectric-transducer (PZT), and the dashed lines are the best fit of the coincidence rates. (**a**) Is the results for photon pairs directly output from the source, (**b**) and (**c**) are the results when the signal photons are distributed over the FMF in *LP*_01_ mode and *LP*_11a_ mode, respectively.

For comparison, we first measure the quality of entangled photon pairs directly output from the source. The data points in Fig. 3(a) show the measured single channel photon count rates and coincidence count rate as a function of ${V}_{PZT}$. One sees the single channel count rates are constant while the coincidence rate changes sinusoidally when ${V}_{PZT}$ is varied. The dashed curve is a best fit of the measured data of coincidence rate to sine-function, which gives a visibility of 95 ± 2%. We then measure the entanglement after the signal photons are distributed over the FMF in the *LP*_01_ mode. The polarization state of the photons input into the FMF is not intentionally controlled. As shown in Fig. 3(b), the single channel rate of the signal photons experiences a decline due to losses induced by transmission and mode converters, but is still constant when ${V}_{PZT}$ is varied. The measured visibility of coincidence counts is 95 ± 3%, showing the degree of entanglement is maintained well. Figure 3(c) shows the results when the signal photons are distributed in the *LP*_11*a*_ mode. Again, no intentionally polarization controlling is applied to the photons. From the results, we find the visibility of coincidence counts is 93 ± 3% and no obvious variation is observed in the single channel rates. Taking the measurement uncertainties into account, one sees that there is no significant decline of the visibility for this case.

The results show the time-bin entanglement is very robust when being distributed over FMF due to the de-coupling between temporal and spatial degrees of freedom. Moreover, since the time delay between the two time slots (1.5 ns in our case) is far less than the variation time of thermal and mechanical drifts of FMF which is in the order of millisecond, the time-bin entangled photon pairs are far less susceptible to the thermal and mechanical drifts.

### Distribution of polarization entangled photon pairs over FMF

**Figure 4:**
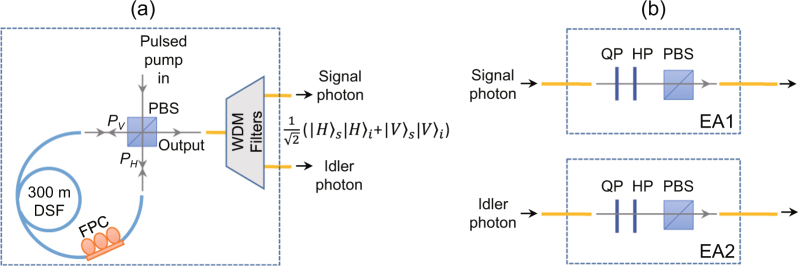
(**a**) Polarization entangled photon pair source and (**b**) entanglement analyzers (EAs). PBS, polarization beam splitter; DSF, dispersion shifted fiber; FPC, fiber polarization controller; WDM filters, wavelength-division multiplexing filters; QP, quarter-wave plate; HP, half-wave plate.

We then experimentally distribute polarization entangled photon pairs over the FMF. The polarization entangled photon pair source is shown in Fig. 4(a), which is also based on pulse pumped SFWM process in a 300-m-long DSF (see ref.^18^ for detailed information). The pump pulse, DSF, and WDM filters of the source are the same as that in Fig. 2(a). As shown in Fig. 4(a), the pump pulses are decomposed into horizontally and vertically polarized components, ${P}_{H}$ and ${P}_{V}$, by the polarization beam splitter (PBS). ${P}_{H}$ propagating in the DSF with clockwise direction generates correlated photon pairs $|H{\rangle }_{s}|H{\rangle }_{i}$, while ${P}_{V}$ propagating with counter-clockwise direction generates photon pairs $|V{\rangle }_{s}|V{\rangle }_{i}$. By adjusting the fiber polarization controller (FPC), $|H{\rangle }_{s}|H{\rangle }_{i}$ and $|V{\rangle }_{s}|V{\rangle }_{i}$ are output from the same port of PBS and coherently add up. Thus the polarization entangled state $|{\rm{\Psi }}\rangle =\frac{1}{\sqrt{2}}{(|H\rangle }_{s}|H{\rangle }_{i}+{e}^{i{\rm{\varphi }}}|V{\rangle }_{s}|V{\rangle }_{i})$ is produced, where $\varphi$ is the accumulated relative phase between $|H{\rangle }_{s}|H{\rangle }_{i}$ and $|V{\rangle }_{s}|V{\rangle }_{i}$ in the process. In the experiment, we set $\varphi =0$ by launching the pump linearly polarized along 45° into the PBS. The polarization EAs are illustrated in Fig. 4(b). Each EA consists of a QP, an HP, and a PBS, and the function of QP and HP is to adjust the angle of EA. By fixing the angle of EA2 at 45° and adjusting the relative angle of EA1 and EA2, ${\rm{\Delta }}\theta$, the fringe of coincidence count rate can be obtained, and the measured coincidence count rate can be expressed as $R\propto 0.5+0.5\,\cos \,\varphi \,\cos \,2{\rm{\Delta }}\theta$.

**Figure 5:**
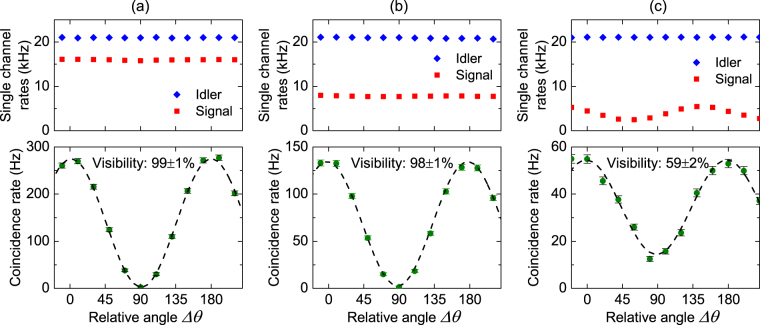
Measured single channel count rates and coincidence count rates of polarization entangled photon pairs. The data are plotted as functions of the relative angle of the polarization entanglement analyzers, and the dashed lines are the best fit of the coincidence rates. (**a**) Is the results for photon pairs directly output from the source, (**b**) and (**c**) are the results when the signal photons are distributed over the FMF in *LP*_01_ mode and *LP*_11*a*_ mode, respectively.

Again we first measure the quality of the polarization entangled photon pairs from the source. The data points in Fig. 5(a) shows the measured single channel count rates and coincidence count rate as a function of ${\rm{\Delta }}\theta$. From Fig. 5(a), one sees the single channel count rates are constant, while the coincidence count rate varies with ${\rm{\Delta }}\theta$. The dashed curve is the best fit of the coincidence count rate, which corresponds to a visibility of 99 ± 1%. From the high visibility of the fringe and the dip We then measure the entanglement after propagating the signal photons over the FMF. Figure 5(b) shows the results when signal photons are distributed over the FMF in *LP*_01_ mode. One sees the single channel rate of signal photons declines due to losses induced by transmission and mode converters, but still remain constant. The visibility of coincidence counts is found to be 98 ± 1%, showing the degree of entanglement is well maintained.

Unfortunately, the situation is quite different when the signal photons are distributed over the FMF in *LP*_11*a*_ mode. As shown in Fig. 5(c), there is a variation in the single channel rate of signal photons and the visibility of coincidence rates reduces to 59 ± 1%. The variation in the single channel rate indicates the mode coupling of polarization states so that the transmitted state is no longer the input entangled states, leading to the decrease of the degree of entanglement. To understand the mode coupling better, we next characterize in detail the transmission properties of various modes in FMF.

### Characterization of mode coupling effect in few mode fiber

**Figure 6:**
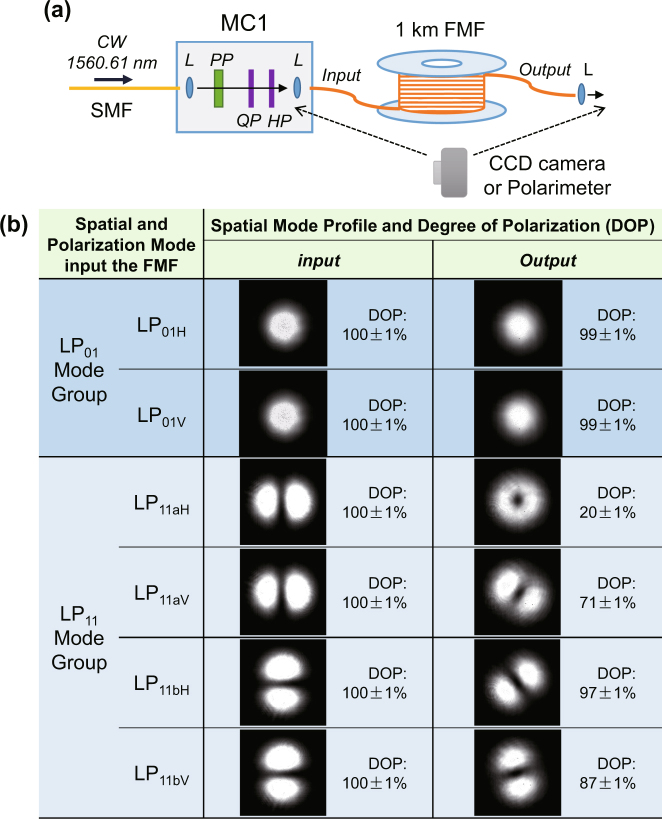
(**a**) Setup for characterization of mode coupling effect in the 1-km-long few-mode fiber (FMF). MC, mode convertor; L, lens; PP, phase plate; QP, quarter-wave plate, HP, half-wave plate. (**b**) Measured spatial mode profiles and degrees of polarization (DOPs) at the input and output ports of FMF.

When distributed in SMF, the de-coherence of polarization entanglement is mainly due to the polarization mode dispersion and polarization mode coupling^19^. In the case of FMF, however, the existence of multiple spatial modes makes the situation more complicated. This is mainly due to the mode coupling effect among different spatial modes. The inset of Fig. 1 shows the first three spatial modes that are relevant to this study. We characterize the mode coupling effect in the 1-km-long FMF by using a 1560.61 nm continuous wave (CW) laser light with a setup shown in Fig. 6(a). By adjusting MC1, we can send the CW light into FMF in six different modes, including two degenerate modes of the *LP*_01_ mode group (*LP*_01*H*_ and *LP*_01*V*_) and four degenerate modes of the *LP*_11_ mode group ($L{P}_{11aH}$, $L{P}_{11aV}$, $L{P}_{11bH}$, and $L{P}_{11bV}$). Here the subscripts *H* and *V* denote the horizontal and vertical polarizations while the subscripts *a* and *b* denote the two degenerate spatial modes having mode profiles with a mutual rotation of 90° (see inset of Fig. 1). For each mode, we measure the spatial profile and degree of polarization (DOP) at the input and output ports of the FMF by using a CCD camera and a polarimeter. The results are shown in Fig. 6(b).

It can be seen that the input spatial profiles and DOPs of each mode are almost ideal, which indicates that MC1 functions as designed. But situations of the outputs are different for the *LP*_01_ and *LP*_11_ mode groups. The output *LP*_01_ modes maintain their mode profiles and DOPs well after transmission. But for *LP*_11_ modes, the output mode profiles are no longer in the original input shapes and the DOPs decline as well. It can be seen that the transmitted $L{P}_{11aH},L{P}_{11aV}$ modes change more drastically than $L{P}_{11bH},L{P}_{11bV}$ with the $L{P}_{11bH}$ and $L{P}_{11bV}$ modes maintaining generally the two-lobe mode profiles but rotated in angles. The results in Fig. 6(b) show that the mode coupling between *LP*_01_ and *LP*_11_ mode groups are negligibly small in our FMF (evidenced by the well maintained *LP*_01_ modes), which is due to the relatively large difference of propagation constants between the two mode groups. However, coupling among the four degenerate modes of *LP*_11_ mode group is quite significant in our FMF.

Assuming the four degenerate modes in *LP*_11_ mode group are linearly coupled in FMF, we can describe the coupling effect in matrix format as

1 $$(\begin{array}{c}{O}_{aH}\\ {O}_{aV}\\ {O}_{bH}\\ {O}_{bV}\end{array})={M}(\begin{array}{c}{I}_{aH}\\ {I}_{aV}\\ {I}_{bH}\\ {I}_{bV}\end{array})$$

where *I*_*ij*_ ($i=a,b,j=H,V$) represents the amplitude of $L{P}_{ij}$ mode at the input port of FMF and ${O}_{ij}$ ($i=a,b,j=H,V$) represents the amplitude of $L{P}_{ij}$ mode at the output port of FMF. ***M*** is a 4 × 4 unitary matrix for lossless transmission with non-zero off diagonal elements. In this picture, for the input of one particular degenerate mode of *LP*_11_ mode group, the corresponding output is a superposition of the four degenerate modes. Therefore, the outputs of two orthogonally polarized input modes is no longer orthogonal.

From the discussion above, it can be seen that the mode coupling effect can be a serious problem when polarization entanglement is distributed over FMF in *LP*_11_ mode. The output mode profiles of the two orthogonally polarized photons are different (see the profiles of $L{P}_{11aH}$ and $L{P}_{11aV}$ in Fig. 6(b)), therefore, no particular angle is set for the phase plate in mode converter at output port of FMF. We also note that by changing the angle of the phase plate, the variation of single channel count rate of signal channel and the interference visibility of coincidence are changed accordingly. Results show the quality of polarization entangled photon pairs experiences significant decline when the photon pairs are distributed in *LP*_11_ mode. However, situation is different for other kinds of entanglement. As shown earlier, the time-bin entanglement is more robust when being distributed in FMF, because the temporal degree of freedom is independent of the polarization degree and is thus immune to polarization mode dispersion and polarization mode coupling. *LP*_01_ modes are also decoupled from *LP*_11_ modes so that entanglement distribution in *LP*_01_ modes is not affected.

### Recovery of polarization entanglement by using linear optics

The polarization coupling effect in *LP*_11_ modes needs to be dealt with before FMF can be used for entanglement distribution. In the application to the traditional optical communication, The mode coupling effect in *LP*_11_ mode group can be overcome by determining the coupling matrix ***M*** in Eq. (1) and inverting its function, which can be realized by either electronic or optical techniques. Through the electronic technique, ***M*** is determined and inverted by coherently and synchronously detecting the output signals in different modes, then digitalizing and processing the detected signals by digital signal processing^20^. However, this technique cannot be employed for entangled photon pairs because the photons are detected by on-off single photon detectors and no phase information of ***M*** can be obtained.

As to the optical solutions, it is hard to find an optical element that can be controlled to function as the inverted unitary matrix ***M*** ^−1^. One possible way is to employ the nonlinear optical processes, such as sum frequency generation (SFG), to realize a spatial-mode-selective frequency conversion^21^. The unitary nature of SFG allows it to preserve the quantum nature of the optical fields in the conversion process. However, the efficiency of the process needs to be improved and the cross-talk and noise of this process need to be further characterized before the quantum applications.

**Figure 7:**
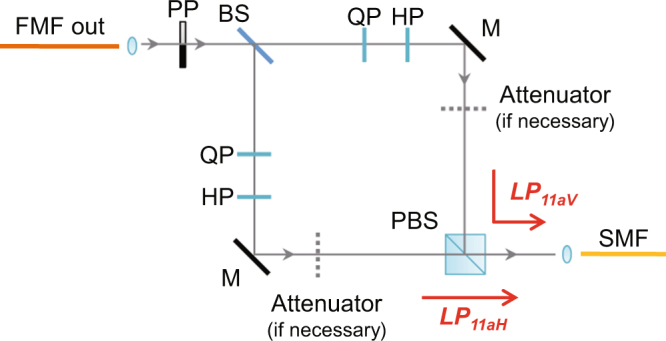
Setup for recovery of polarization entanglement by using linear optics. FMF, few-mode fiber; SMF, single-mode fiber; PP, phase plate; QP, quarter-wave plate; HP, half-wave plate; M, mirror; BS, beam splitter; PBS, polarization beam splitter.

Here we propose and demonstrate a method that is based on linear optics to recover the polarization orthogonality destroyed by mode coupling. The key is to use mode selection method to project the 4-dimensional space into a 2-dimensional space which can be handled with linear optics. The setup of our method is shown in Fig. 7. The output of FMF first passes through a PP for mode selection and then is split into two beams by a 50:50 beam splitter. Two sets of QP and HP are placed in the two arms, respectively. Then the two beams are combined by a PBS and collected into SMF. The path difference of the upper and lower arms is set to be zero. Note the angles of PP and PBS define the $L{P}_{11aH}$ and $L{P}_{11aV}$ modes at the output port of FMF for mode selection. $L{P}_{11bH}$ and $L{P}_{11bV}$ modes are rejected from coupling into the SMF.

To explain the working principle of our setup, we consider the input modes $L{P}_{11aH}$ and $L{P}_{11aV}$ only in Eq. (1) because of the mode projection achieved by the phase plate. Then Eq. (1) is reduced to

2 $$(\begin{array}{c}{O}_{aH}\\ {O}_{aV}\end{array})={\boldsymbol{W}}(\begin{array}{c}{I}_{aH}\\ {I}_{aV}\end{array})=(\begin{array}{cc}{w}_{11} & {w}_{12}\\ {w}_{21} & {w}_{22}\end{array})(\begin{array}{c}{I}_{aH}\\ {I}_{aV}\end{array}),$$

where ***W*** is the 2 × 2 matrix of the upper left part of the 4 × 4 matrix ***M***. Because of the projection, ***W*** is not unitary.Now we consider separately the upper and lower arms in Fig. 7. For the upper arm, we adjust the corresponding QP and HP to make them functioning as the following unitary matrix

3 $${(|{w}_{11}{|}^{2}+|{w}_{21}{|}^{2})}^{-\frac{1}{2}}(\begin{array}{cc}{w}_{11}^{\ast } & {w}_{21}^{\ast }\\ -{w}_{21} & {w}_{11}\end{array})\mathrm{.}$$

So the angles of the QP and HP are configured according to the elements of ***W***. By adding this matrix to the right-hand side of Eq. 2, we arrive

4 $${(\begin{array}{c}{O}_{aH}\\ {O}_{aV}\end{array})}_{upper}={(|{w}_{11}{|}^{2}+|{w}_{21}{|}^{2})}^{-\frac{1}{2}}(\begin{array}{cc}{w}_{11}^{\ast } & {w}_{21}^{\ast }\\ -{w}_{21} & {w}_{11}\end{array})(\begin{array}{cc}{w}_{11} & {w}_{12}\\ {w}_{21} & {w}_{22}\end{array})(\begin{array}{c}{I}_{aH}\\ {I}_{aV}\end{array}),$$

from which one can find ${O}_{aV}={(|{w}_{11}{|}^{2}+|{w}_{21}{|}^{2})}^{-\frac{1}{2}}({w}_{11}{w}_{22}-{w}_{12}{w}_{21}){I}_{aV}$. Thus the one to one function between ${O}_{aV}$ and ${I}_{aV}$ has been established. Similarly, for the lower arm, the corresponding QP and HP are also properly adjusted to realize the following evolution

5 $${(\begin{array}{c}{O}_{aH}\\ {O}_{aV}\end{array})}_{lower}={(|{w}_{12}{|}^{2}+|{w}_{22}{|}^{2})}^{-\frac{1}{2}}(\begin{array}{cc}{w}_{22} & -{w}_{12}\\ {w}_{12}^{\ast } & {w}_{22}^{\ast }\end{array})(\begin{array}{cc}{w}_{11} & {w}_{12}\\ {w}_{21} & {w}_{22}\end{array})(\begin{array}{c}{I}_{aH}\\ {I}_{aV}\end{array})\mathrm{.}$$

So that the one to one function ${O}_{aH}={(|{w}_{12}{|}^{2}+|{w}_{22}{|}^{2})}^{-\frac{1}{2}}({w}_{11}{w}_{22}-{w}_{12}{w}_{21}){I}_{aH}$ is also established for the lower arm.The PBS is employed to combine ${O}_{aV}$ of the upper arm and and ${O}_{aH}$ of the lower arm. In the output port of PBS, we have

6 $${(\begin{array}{c}{O}_{aH}\\ {O}_{aV}\end{array})}_{c}=({w}_{11}{w}_{22}-{w}_{12}{w}_{21})(\begin{array}{cc}{(|{w}_{12}{|}^{2}+|{w}_{22}{|}^{2})}^{-\frac{1}{2}} & 0\\ 0 & {(|{w}_{11}{|}^{2}+|{w}_{21}{|}^{2})}^{-\frac{1}{2}}\end{array})(\begin{array}{c}{I}_{aH}\\ {I}_{aV}\end{array})\mathrm{.}$$

Since ${(|{w}_{11}{|}^{2}+|{w}_{21}{|}^{2})}^{-\frac{1}{2}}$ and ${(|{w}_{12}{|}^{2}+|{w}_{22}{|}^{2})}^{-\frac{1}{2}}$ are not necessarily equal, we add an attenuation to either of the arms to make the two quantities equal to *m*, where $m=\,{\rm{\min }}\,\{(|{w}_{11}{|}^{2}+|{w}_{21}{|}^{2}{)}^{-\frac{1}{2}},(|{w}_{12}{|}^{2}+|{w}_{22}{|}^{2}{)}^{-\frac{1}{2}}\}$. So the amplitudes of ${O}_{aH}$ and ${O}_{aV}$ are balanced and the final evolution from the input of FMF to the output of the PBS can be written as

7 $${(\begin{array}{c}{O}_{aH}\\ {O}_{aV}\end{array})}_{c}=m({w}_{11}{w}_{22}-{w}_{12}{w}_{21})(\begin{array}{cc}1 & 0\\ 0 & 1\end{array})(\begin{array}{c}{I}_{aH}\\ {I}_{aV}\end{array})\mathrm{.}$$

Thus, the orthogonality of the input modes $L{P}_{11aH}$ and $L{P}_{11aV}$ is successfully recovered.

It should be pointed out there are two limitations of our method. The first one is the loss of the system can be high, and it depends not only on the transmission and coupling efficiencies of the optical components but also on the elements of ***W***. However, loss does not affect the quality of the polarization entangled photon pairs. The second one is that our method does not work if ***W*** is not invertible (or equivalently ${w}_{11}{w}_{22}-{w}_{12}{w}_{21}=0$ is satisfied). But this is a rare case. Moreover, our recovery method can also be applied to other high-order transverse mode groups which contain spatial degenerate modes, as long as proper mode conversion components, such as phase plates or spatial light modulators, are available.

We then experimentally test the performance of our method. The experimental setup is the same as that shown in Figs 1 and 4 except replacing MC2 by the recovery setup shown in Fig. 7. Before distributing entangled photons, the recovery setup needs to be configured first. We send a CW light at 1560.61 nm into the FMF in $L{P}_{11aH}$ mode and rotate the QP and HP in the upper arm of the recovery setup to ensure no light is reflected by the PBS. Then we change the input light to $L{P}_{11aV}$ and adjust the QP and HP in the lower arm to ensure no light passes through the PBS. Therefore, only the input mode $L{P}_{11aV}$ ($L{P}_{11aH}$) can travel through the upper (lower) arm to the PBS output. Finally, we compare the intensities from the two arms and insert a proper attenuator into one arm to make the two intensities equal. After the configuration, the total loss of the recovery setup in our experiment is about 8 dB.

**Figure 8:**
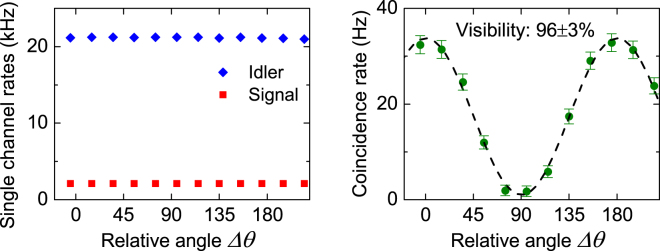
Measured single channel count rates and coincidence count rates of polarization entangled photon pairs. The results are obtained when signal photons is distributed over FMF in $L{P}_{11a}$ mode and the recovery setup is employed. The data are plotted as functions of the relative angle of the polarization analyzers, and the dashed line is the best fit of the coincidence rates.

We now are ready to distribute entangled photon pairs. The experimental results are shown in Fig. 8, which are obtained when the signal photons is distributed over FMF in $L{P}_{11a}$ mode and the recovery setup is employed. It can be seen that the single channel counts of signal photons experience a large drop due to the loss of recovery setup. But the visibility of coincidence count is recovered to 96 ± 3%, which is much higher than the case without the recovery setup (Fig. 5(c)). This result shows the effectiveness of our method. We note that the recovered visibility is slightly lower than the visibility of photon pairs directly from the source (Fig. 5(a)). We believe it is because of the imperfections in the polarization control and power balance when configuring the recovery setup.

## Conclusion

In summary, we have studied the distribution of entangled photon pairs over FMF. We are able to respectively distribute the time-bin entangled and polarization entangled photon pairs over a 1-km-long FMF in either *LP*_01_ or *LP*_11_ spatial modes even though special care has to be paid for the polarization case due to the mode coupling effect in the FMF. Results show the time-bin entanglement is robust when distributed in either spatial modes, since it is far less susceptible to the mode coupling effect. In contrast, the effect of mode coupling on polarization entanglement depends on the employed spatial modes. The polarization entanglement is well maintained after being transmitted in *LP*_01_ mode. But when the polarization entanglement is transmitted in *LP*_11_ modes which consist of spatial degenerate modes $L{P}_{11a}$ and $L{P}_{11b}$, the quality of the entanglement will decrease due to the mode coupling between the degenerate modes in the mode group. We therefore conclude that higher order mode groups containing multiple degenerate spatial modes, such as $L{P}_{xy}$ (x, y = 1, 2, 3…), are not suitable for the distribution of polarization entanglement. For relatively lower number of degeneracy of *LP*_11_ modes, we also propose and demonstrate a method for the recovery of polarization entanglement after it is affected by the mode coupling effect. The method is based on linear optics and mode projection and its effectiveness is experimentally verified. For multi-mode fiber in which much more spatial modes are involved, the mode coupling effect is more complicated even if the entanglement is distributed along the principal modes^22^. For polarization entanglement, as long as the mode coupling effect can be generally described by the coupling matrix, our projecting and recombining method will be still effective to reconstruct the polarization orthogonality. Our investigation is useful for the long distance high capacity quantum communication based on FMF network.

## Methods

We describe the single photon detection and data acquisition system shown in Fig. 1. The two SPDs are based on InGaAs avalanche photo diodes working in the gated Geiger mode. The 41 MHz gate pulses originate from a mode-locked laser and are properly delayed to coincide with the arrival of the signal and idler photons at the SPDs. Since the signal and idler photons travel different distances before being detected, we acquire all the detection events of the SPDs by using a computer-based data acquisition system. Then we obtain the coincidence count rates of signal and idler photons produced within the same pump pulse, and the accidental coincidence count rate of signal and idler photons produced respectively by different pump pulses.

The coincidence count rates in Figs 3, 5 and 8 are the results after subtracting the accidental coincidence. The error bar of each data point represents one standard deviation error of the measurement. We fit each set of coincidence data with a sine-function by utilizing the orthogonal distance regression algorithm. The visibility of the fringe is one of the fitting parameters. Once the fit is converged, a fringe visibility with one standard deviation error can be obtained.

In order to minimize the influences of the thermal and mechanical drifts of the FMF, we fix the FMF on an optical table and the experiments are conducted in air-conditioned room. By doing so, the mode profile and polarization state outputs from FMF can be kept constant for hours. Since it takes less than five minutes to obtain a set of data, the influences of the drifts can be negligible. However, for long term measurements, the drifts could be significant, but the problem can be solved by sending probe beam at different wavelength into transmission fiber and accordingly set up the feedback control system.
